# Sequence Composition and Evolution of Mammalian B Chromosomes

**DOI:** 10.3390/genes9100490

**Published:** 2018-10-10

**Authors:** Nikolay B. Rubtsov, Yury M. Borisov

**Affiliations:** 1 The Federal Research Center Institute of Cytology and Genetics SB RAS, Lavrentiev Ave. 10, Novosibirsk 630090, Russia; 2 Novosibirsk State University, Pirogova Str. 2, Novosibirsk 630090, Russia; 3 Severtzov Institute of Ecology and Evolution, Russia Academy of Sciences, Leninsky Pr. 33, Moscow 119071, Russia; boriss-spb@yandex.ru

**Keywords:** B chromosomes, karyotypes, genome evolution, interphase nucleus, mammals, genes, repetitive DNA, transcription of heterochromatin

## Abstract

B chromosomes (Bs) revealed more than a hundred years ago remain to be some of the most mysterious elements of the eukaryotic genome. Their origin and evolution, DNA composition, transcriptional activity, impact on adaptiveness, behavior in meiosis, and transfer to the next generation require intensive investigations using modern methods. Over the past years, new experimental techniques have been applied and helped us gain a deeper insight into the nature of Bs. Here, we consider mammalian Bs, taking into account data on their DNA sequencing, transcriptional activity, positions in nuclei of somatic and meiotic cells, and impact on genome functioning. Comparative cytogenetics of Bs suggests the existence of different mechanisms of their formation and evolution. Due to the long and complicated evolvement of Bs, the similarity of their morphology could be explained by the similar mechanisms involved in their development while the difference between Bs even of the same origin could appear due to their positioning at different stages of their evolution. A complex analysis of their DNA composition and other features is required to clarify the origin and evolutionary history of Bs in the species studied. The intraspecific diversity of Bs makes this analysis a very important element of B chromosome studies.

## 1. Introduction

The story of studying and describing B chromosomes (Bs) dates back to 1907, when Edmund Wilson, working on hemipteran chromosomes, noticed those that appeared to be additional to the main karyotype and were present only in a fraction of individuals [1]. However, the term ‘B chromosome’ was only established 11 years later. In 1928, Lowell Fitz Randolph working on variation in maize chromosomes proposed to call stable chromosomes of the standard complement ‘A chromosomes’, and those coming additional to the standard complement and being variable in number and morphology, ‘B chromosomes’ [2]. This term has since become widely recognized and used, even though it causes certain issues. It is considered that Bs are the chromosomes that exist in addition to the chromosomes of the main karyotype and are present only in some of the individuals of any given species. They have been found in karyotypes of species in most of the large taxa of multicellular organisms. Bs have not yet been reported in birds whose chromosomes are similar to mammalian ones showing G and R-bands. Germline-restricted chromosomes revealed in zebra and Bengalese finches [3,4,5] are sometimes named Bs, however, they do not fulfill the requirements of the given definition since they are obligatory elements of germline cell karyotype. It is possible that Bs do occur in some avian species but happen to be camouflaged by numerous microchromosomes precluding an accurate count of chromosome number on metaphase plates. It cannot be excluded either that their absence in avian karyotypes is due to strict negative selection against ‘superfluous’ DNA in the evolution of small avian genome [6]. 

B chromosomes are not necessary for normal development or reproduction, although in some species they are present in most individuals’ karyotypes [7,8]. Most DNA in Bs consists of repeated elements. Often, they can be arranged in arrays of tandem repeats. In some species, Bs contained also clusters of ribosomal DNA (rDNA) forming nucleolus organizer regions (NORs) detected with AgNOR silver staining [9] or regions enriched for repeat homologous to rDNA [10,11]. Sequences identified in Bs as being homologous to unique sequences of the main genome probably occur in multiple copies too [12,13]. As DNA composition and morphology of Bs are extremely diverse within a single species, population and even individual animals [8,14], it is very difficult to say how fast they are changing throughout generations. Nevertheless, divergence time was estimated for a few sequences revealed in Bs of some grasshoppers (0.75 Myr) [15] and in Bs of some rye species (1.1–1.3 Myr) [16]. 

Of special interest is the meiotic behavior of Bs and their effects on conjugation, chiasma formation and A chromosome crossover. It has been demonstrated that their presence can lead to changes in chiasma frequency and recombination rate variation [17,18]. The question as to whether Bs are genomic parasites or they are precious items in the evolutionary inventory has yet to be answered. Probably, some of Bs are just genomic parasites while other Bs could contain elements that are able to develop in the future to new genes or could modify some genetic processes. It has been hypothesized that variation in the number and morphology of Bs may help some species to adapt to special environments [19] or at least changes the phenotypic features [20,21,22].

While Bs in different species look similar, due to Bs sequencing and some other results obtained by modern experimental techniques we make an attempt to find and follow differences between these Bs by taking into account particularities of the main genome of their carriers. The comparative analysis of specific features of mammalian Bs and similar features of mammalian A chromosomes was performed with respect to specific distribution of genes in chromosome regions, their replication timing, transcriptional regulation, and preferred places they occupy in the nuclei of somatic and meiotic cells. Despite of impressive progress in B chromosome studies taking place during the last years, it should be mentioned that the knowledge of mammalian Bs remains scarce and their organization, significance and evolution should be discussed once again following the sequencing of new Bs, description of their transcriptional activity, and intensive studies of their other features. 

## 2. Mammalian Genomes: Organization and Diversity

The genome size in mammals shows little interspecific variation [6,23]. There is no evidence for a whole-genome duplication event in mammalian genome evolution [24]. In mammals, only a limited number of tissues are composed of polyploid cells. The hypothesis that the vizcacha rat might be tetraploid [25] failed to be confirmed by later efforts [26]. For most of its part, variation in mammalian genome size is explained by differences in the volume of repeated DNA, although duplications and even amplifications of a small ‘unique’ portion of the genome are known to have taken place in various phylogenetic lines of mammals [27]. In this respect, Bs are special entities in mammalian genome evolution providing in some species large variation of constitutive heterochromatin volume [18]. 

In humans, size variation of constitutive heterochromatin (C-positive chromosome regions) in A chromosomes usually is not associated with developmental abnormalities and there is few data on the influence of the size of heterochromatic regions on adaptiveness or other phenotypical features. From work on this topic, we would like to mention a study devoted to comparison of the heterochromatic region size in chromosomes of individuals from human populations with a long record of living in contrasting climatic conditions (the Far North, the subtropics and tropics, lowlands and uplands) [28,29]. The results of these studies suggest that at least some of the size variation of heterochromatic regions may be of adaptive value as a species is exploring a new ecological niche to settle in. In some species, Bs provide considerable variation of the heterochromatin volume [18], nevertheless, there is only a few data on the influence of their number on phenotype of their carrier [20,21]. 

The euchromatic regions of mammalian chromosomes consist of G and R-bands. These bands differ in many ways including replication timing profiles and gene transcription activity. Being mostly heterochromatic, Bs for a long time had been considered as elements containing no genes and transcriptionally inert. However, DNA fragments homologous to different genes were revealed recently in Bs of some species despite their C-positive pattern [30,31]. Furthermore, transcription of some of them was shown [32]. We should also mention that most of Bs are highly heterochromatic but in some species G-banding of Bs was described [33,34,35]. 

DNA fragments of Bs homologous to genes of main genome could bring into total genome additional copies of ‘unique’ DNA fragments. The significance of copy number variations (CNV) of different DNA regions were studied in numerous investigations of patient genomes performed with whole genome sequencing and microarray-based comparative genome hybridization [36]. It was shown that the size of the regions varied in copy numbers could be strongly different in size. Some of them have been found to exert a considerable effect on the phenotype, while the others have not been found to be associated with any developmental pathology [37,38]. Taking together, these data encourage thorough comparative analysis of the regions containing homologous genes in A and B chromosomes. Application of Hi-C technology [39] for analysis of interaction of these B chromosome regions with other elements of main genome can be efficient approach for such comparative studies. 

## 3. Mammalian B Chromosomes: Prevalence and DNA Content

Bs can add to the main genome a considerable amount of DNA homologous both to its repeated and unique sequences, including the introns and exons of various genes and sometimes probably entire genes together with their flanking sequences [13,30,31,40]. Up to now, Bs have been found in more than 70 mammalian species [7] and the number is growing constantly. Furthermore, because Bs can be found in different species in different numbers, it does not seem feasible to count how many mammalian species contain them. While some species have them in most individuals, others have them only in a few. Moreover, differences in the frequency of the Bs are known even between populations. For example, Bs are present in most (over 90%) Korean field mice dwelling in Siberia and the Russian Far East. A cell may contain up to thirty Bs [8,41,42]. At the same time, no Bs have been found in these mice’s conspecifics inhabiting Sakhalin and Stenin Island [42]. That is, if the karyotyping of a small number of individuals from several populations fails to reveal Bs, it is, to say the least, precautious to conclude that Bs do not occur in this species at all.

A lot of relevance lies with the studies of small supernumerary marker chromosomes (SMCs) in humans. At the First B chromosome Conference held in 1993 in Madrid, the following definition of the B chromosome was given: “A dispensable supernumerary chromosome that does not recombine with the A chromosomes and follows its own evolutionary pathway” [10]. Small SMCs are dispensable supernumerary elements of human karyotype. In contrast to majority of Bs, human small SMCs are usually exact copy of the original chromosome region [43,44]. The question on their recombination with A chromosomes remains open but it is possible suggesting that the frequency of such recombination is at least diminished. It is impossible to say that they already “follow their own evolutionary pathway” but they are probably similar to Bs on the initial stage of their evolution. It cannot be excluded also that there are small SMCs that have undergone some additional changes and have thus become almost full-fledged Bs; however, the example of human small SMC with additional changes has yet to be found.

Thus, there is little wonder why the question as to whether small SMCs in humans should be regarded as Bs was addressed separately [45]. Like many Bs, human small SMCs normally contain pericentric C-positive regions of A chromosomes. The presence of additional euchromatic regions in them could lead to developmental abnormalities [44,46,47]. Composition of small SMCs derived from human chromosomes 15 and 22 is rather precisely determined, while the euchromatic part of other SMC composition often remains an open question. This inaccuracy in identifying breakpoint positions, and consequently, in determining DNA content of small SMCs makes it difficult to predict what clinical effects they may have [48]. Furthermore, SMCs containing small euchromatic regions next to pericentric heterochromatin do not cause development abnormalities [49]. We suppose that human small SMC might not be regarded as Bs but the mechanism of their formation could be similar to those one of proto-B chromosomes. The study of human small SMC can provide useful data to understand chromosome rearrangements leading to B chromosome formation and its DNA content on initial stage of their evolution. 

Analysis of B chromosome composition usually reveals DNA homologous to the pericentric region of A chromosomes, this DNA being often multiply amplified forms of additional extended arms of Bs [8,9,14]. Sometimes a more detailed analysis of Bs reveals DNA that is atypical for pericentric regions. Bs were found to contain additional types of repeats [8,13,31] and sequences homologous to unique DNA fragments in the main genome, including gene fragments and probably entire genes accompanied with adjacent sequences [31,40]. Moreover, data suggest that Bs may contain extended DNA regions—up to several millions of base pairs in size—homologous to euchromatic regions in A chromosomes [31]. The first evidence that those regions can be found in Bs was discovered as a by-product of a comparative cytogenetic analysis performed on red fox and raccoon dog chromosomes, using fluorescence in situ hybridization (FISH) with DNA probes designed from canine bacterial artificial chromosomes containing *C-KIT* and its adjacent sequences [30]. Red fox (*Vulpes vulpes*) and raccoon dog (*Nyctereutes procyonoides*) diverged about 12.5 million years ago; however, Bs in both of them contain homologous DNA regions [40].

**Figure 1:**
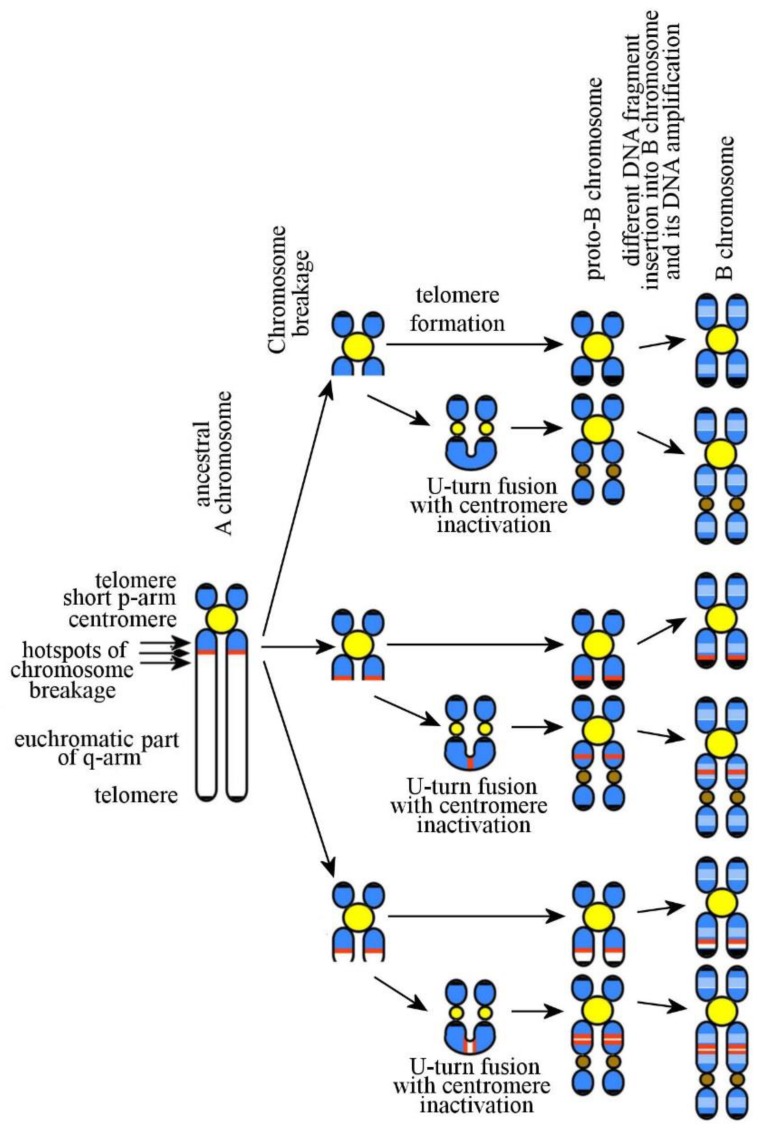
The B chromosomes (Bs) result from a chromosomal breakage in a pericentric heterochromatic region, a duplicon cluster, and a proximal euchromatic region. Euchromatic regions and insertions of euchromatic material in Bs are white; heterochromatic regions and insertions of heterochromatic material in Bs are blue; clusters of duplicons are red; active centromeres are yellow; inactive centromeres are brown; telomeres at the chromosome termini are black.

Do these Bs share a common ancestor, or did they emerge independently, with genes being present in them solely because of a high frequency of inclusions of the DNA fragments containing these genes in Bs? While searching for an answer, it is important to remember that the sequencing and analysis of the human genome have revealed a large number of clusters consisted of duplicated sequences. Some of these duplicons are large and may include whole genes together with their flanking regions [50]. Bs probably are excellent recipients for such duplicated sequences. Another explanation for the presence in Bs of homologous gene copies could include the occurrence in an ancestor of a chromosome containing the genes or their copies in the pericentric region and the chromosomal rearrangement hotspot distal to it. The presence of such A chromosome in karyotype could lead to a high frequency of proto-Bs containing this group of gene copies. Hypothetical mechanisms leading to arising of Bs with different DNA composition are shown in Figure 1. Considering these hypotheses, it is important to keep in mind a complex organization of Bs in different species. Verification of the suggested hypothesis requires the study of their DNA composition and gene content.

Breakthrough in this field is owing to the application of high-throughput sequencing of microdissected or flow-sorted DNA libraries obtained from Bs. This approach was used for studying Bs in six mammalian species [13,31,51,52] and remarkable differences between their gene content and DNA composition were found. The Siberian roe deer (*Capreolus pygargus*) Bs had two regions homologous to those in cattle chromosomes (1.42–1.98 Mbp in total). They contained three genes, while the gray brocket deer (*Mazama gouazoubira*) Bs had 26 such DNA regions (8.28–9.31 Mbp in total) with 34 whole genes and 21 gene fragments, including the proto-oncogenes *С-KIT* and *RET*, of which homologs had earlier been found in canid Bs [52]. There is a large number of mutations that distinguish the homologous sequences of the Bs in the Siberian roe deer, suggesting no strict selection has acted to keep the original DNA sequences in the Bs. By contrast, DNA in the Bs of the gray brocket deer was more homogeneous and more similar to DNA in A chromosomes [52]. Regrettably, the number of individual Bs of deer species that have been studied in detail is too low to discuss the intraspecific DNA heterogeneity in these Bs. We should note that DNA content of Bs in Siberian roe deer and brocket deer was revised recently and the total size of regions homologous to cattle chromosomes was increased from 1.96 Mbp to 2.36 Mbp in Bs of Siberian roe deer and from 9.31 Mbp to 10.46 Mbp in Bs of gray brocket deer [31].

As it was earlier shown, Bs in the raccoon dog (*Nyctereutes procyonoides*) contained at least two types of heterochromatin, interstitial telomere repeats, and DNA fragments homologous to rDNA [53,54,55]. Later, a more detailed analysis of Bs in two subspecies of raccoon dog and in red fox (*V. vulpes*) revealed DNA fragments homologous to C-KIT [30]. It was shown that these Bs also contained the flanking regions [40]. Sequencing of flow-sorted and microdissected DNA libraries of red fox and raccoon dog Bs revealed numerous sequences homologous to DNA fragments located in different A chromosomes of red fox and reference species [31] suggesting that numerous independent insertions could take place during evolution of these Bs or a large duplicon cluster derived from ancestral chromosome was included in initial proto-Bs of canid species. Two overlapping regions revealed in the fox and raccoon dog Bs we consider as argument in favor of the latter suggestion [31].

Other Bs that were intensively studied are the Bs in the Korean field mouse. The studies were started with a comparative analysis of Bs in mice captured at different locations of this species’ habitat using various methods of cytogenetic analysis: From routine morphometry to microdissected DNA library generation followed by FISH on metaphase chromosomes [8,14,41]. 

The results obtained revealed a high diversity of Bs in number, size, repeated sequences, and ancestral chromosomes. Virtually all Bs attributes and properties studied varied in a wide range. The number of Bs in the Korean field mouse (*Apodemus peninsulae*) varies from 0 to 30 [41]. The size of Bs in this species varies from a dot-like chromosome to the largest chromosome in the karyotype [9]. Some of the Bs occur as acrocentrics and some as bi-armed chromosomes [8,41]. Natural populations of mice located in distant geographical regions appear also to be different in the frequency of Bs referred to different morphotypes [8,14].

Differences in the DNA of pericentric regions give reason to believe that some Bs in mice from Siberian populations originated from autosomes, while Bs in mice from Russian Far East are from a sex chromosome [8]. Analysis of chromatin folding has revealed both C-positive regions in Bs and regions that remain poorly condensed even in the late metaphase [9]. The latter regions cannot be visualized by Giemsa or 4′,6-Diamidine-2′-phenylindole dihydrochloride (DAPI) staining techniques; however, thanks to their specific DNA content, they are reliably visualized and identified by two-color FISH with microdissected DNA probes derived from micro-Bs and the pericentric region of large autosome of this species. As was found, each of the most micro-Bs are composed of a small pericentric region including DNA homologous to DNA in autosomal pericentric heterochromatin and a region(s) consisted of other repeats. The latter regions remain poorly condensed in mitosis. Poorly condensed regions, containing repeats such as these, have also been found in the distal regions of some macro-Bs [9]. These repeats have not been observed clustered in A chromosomes or in the interstitial regions of macro-Bs. Additionally, in some Bs rDNA was detected. In A chromosomes of Korean field mouse, rDNA clusters are in the terminal regions of the long arms of two pairs of autosomes, suggesting that rDNA was inserted into Bs at advance evolutionary stages [9]. The sequencing of microdissected DNA libraries of macro and micro-Bs also suggests multiple insertions of DNA fragments from A chromosomes to developed modern Bs [13]. In the mouse genome, the total size of DNA regions homologous to macro-B DNA was estimated at 9.4 Mbp (without repeated sequences totaling 7296 kbp length), while that of DNA regions homologous to micro-B DNA was 5.5 Mbp (without repeated sequences totaling 3935 kbp length). Bs included regions homologous to DNA in different mouse chromosomes: MMU1 (*Cntnap5a* and *Cntnap5b*), MMU5 (*Vmn2r84-Vmn2r87*), MMU7 (*NLR* genes), MMU9 (*Kif23*), MMU10 (the gene encoding the G protein-coupled receptors active in vomeronasal sensory neurons, and probably *Tespa1*), MMU12, MMU13, and MMU17 (*Cntnap5c*). Regions homologous to DNA in MMU1, MMU5, and MMU9 were present in both macro-B and micro-B, while the others, only in the macro-B. As was expected, the DNA of both Bs was found to be enriched for L1 long interspersed nuclear elements [13,31]. Considering the differences revealed with FISH using microdissected DNA probes on Korean field mouse metaphase chromosomes [8,9], the differences found later in the content of repeated sequences [13,31] were expected. A comparison of DNA content in the Bs of Korean field mouse and yellow-necked mouse (*Apodemus flavicollis*) showed that the Bs of both *Apodemus* species contain DNA homologous to the *Vrk1* gene (vaccinia related kinase 1) in mice [31,56]. However, the margins of *Vrk1* region differed in Bs of the studied species, suggesting independent insertions of *Vrk1*-containing DNA fragments into these Bs [13]. 

It should be noted that the genes homologous to sequences found in the Bs of the Korean field mouse had previously been associated with evolutionary breakpoint regions in the porcine genome [57]. The same is probably true for Bs of other species [12]. Taking all data together, it is possible to suggest the different reasons for B chromosome enrichment for copies of certain genes: (*i*) these genes are often involved in duplicon cluster formation; (*ii*) they are located in the vicinities of evolutionary breakpoints; (*iii*) there exist positive natural selection for the functional activity of their copies located in Bs; (*iv*) a combination of the above reasons; (*v*) unknown evidence.

To date, gene content in Bs has been determined with high-throughput sequencing of flow-sorted or microdissected libraries in six mammalian species belonging to canids, ruminants, and rodents [31]. Comparative analysis of the list of these genes allowed suggesting that this list is enriched for cell-cycle-related genes, development-related genes, and genes functioning in synapses. Genes belonging to these groups were also found in Bs of non-mammalian lineages: In Bs of cichlid *Lithochromis rubripinnis* morphogene *Ihhb* (Indian hedgehog b) [22]; in cichlid *Astatotilapia latifiscata* genes associated with cell division [58]; in rye *Secale cereal* pseudogenes and regulatory genes [26,59]; in a grasshopper *Eyprepocnemis plorans* five genes involved in cell division [60].

Ribosomal DNA or DNA partially homologous to it is a usual component of many Bs. There are multiple examples of species in which Вs are enriched by DNA fragments homologous to rDNA with or without nucleolus organizer region formation [9,11]. Change of rDNA location within and between the chromosomes of even closely related species has been found in many phylogenetic lines of mammals [61]. It is possible that Bs are recipients of rDNA in transposition and offer good conditions for the amplification of inserted copies. 

Virtually any detailed study of DNA content in Bs using high-throughput sequencing of the DNA libraries of these Bs and other techniques have revealed sequences homologous to gene fragments and quite extended A chromosomal regions [13,31]. To answer the question on similarity or diversity of Bs within species or B chromosome presence in different species, special importance should be given to more extended studies of their DNA content. 

## 4. Transcriptional Activity of DNA in Mammalian B Chromosomes

The finding that Bs contain DNA sequences homologous to genes of the main genome raised a question about their transcriptional activity. In relation to Вs in various species, this question was addressed in detail in recent review [62], allowing us to focus on mammalian Bs. Probably, it would be useful to divide the discussion of this problem in two parts: transcription of DNA homologous to genes of main genome and transcription of repetitive DNA. Gene transcription from Bs can be reliably detected due to differences between the B chromosome gene sequences and homologous A chromosome gene sequences. With reliance on these differences, the transcriptional activity of genes found in Bs of the Siberian roe deer has been demonstrated [32]. Considering the size variation of DNA inserts in Bs and the diversity of their flanking regions, we would like to speculate that the transcriptional activity of genes in Bs may vary considerably. This is consistent with data on the transcriptional activity of genes in Bs in plants and insects [62]. Most mammalian Bs contain extended heterochromatic regions [54,63]. If some genes in B chromosome are close to these regions, their transcriptional activity can be partially or fully suppressed. In our opinion, some data on gene transcription in Bs may represent a record of low-level transcription, which has no effect on normal cell function. This is supported by data from patients with human small SMCs that contain small euchromatic regions next to pericentric heterochromatin [49]. Healthy carriers of small SMCs with euchromatic centromere-near (ECN) imbalances in small (0.3–5 Mbp) euchromatic regions have been revealed. However, the matter of B chromosome gene transcription is far from being clear. There are Bs containing extended C-negative regions. Some of their examples are Bs of the yellow-necked mouse, *A. flavicollis* [64]. Differential display reverse transcription-polymerase chain reaction (DDRT-PCR) was used for comparative analysis of gene expression in these animals with and without B chromosome. The following three complementary DNA (cDNA) fragments with differential expression were revealed: Chaperonin containing TCP-1 subunit 6b (zeta) (*CCT6B*), fragile histidine triad gene (*FHIT*), and a hypothetical gene XP transcript. Their differential expression was confirmed by real-time PCR. The study of DNA content of five Bs in *A. flavicollis* [13,31] revealed DNA fragments homologous to 38 genes. Twenty-nine of them may form parts of functional clusters. The largest number of the genes revealed were those encoding microtubule-associated and cell-cycle gene proteins: *Cenpe* (centromere protein E), *Dync1i2* (dynein cytoplasmic 1 intermediate chain 2), *Mns1* (meiosis-specific nuclear structural protein 1), and *Mapre1* (microtubule-associated protein, RP/EB family, member 1). It should be noted that four of the studied Bs were from the Serbian populations and one was from Russian. The one from the Russian population contained a DNA fragment homologous to a ~300 kbp sequence of house mouse chromosome 9, which the Bs of the animals captured in Serbia did not. The intraspecific differences found in DNA content between Bs complicate the study on transcription of the genes in Bs. Data about the copy numbers of studied genes, DNA methylation and histone modification, regions flanking genes, and distance from heterochromatic regions are required for the correct interpretation of revealed transcriptional activity. 

It would be appropriate to look back at the transcriptional activity of repetitive DNA in C-positive regions. Transcription of pericentric satellite repeats have been observed in a large number of species and have been associated with various processes, including cell proliferation, ontogenesis, cellular differentiation, ageing, stress response and cellular transformation [65,66,67]. Transcription of gene copies located in heterochromatic Bs could be a result of their involvement in the general process of repeat transcription [66,68,69], probably taking place in the Bs, or it could represent a specific process of major importance for perfect ontogenesis. 

Unfortunately, little is known about the transcription of repeated sequences in heterochromatic regions of mammalian chromosomes. Transcription of pericentric heterochromatin in mouse embryonic fibroblasts is one of the examples of repetitive DNA studied in detail [70]. Here, the first wave of major satellite transcription was observed in late G_1_ phase, peaking in early S phase. The transcripts were heterogeneous, varying in length from 1000 to more than 8000 bp. It is possible that this stage-dependent transcription is associated with the preparation of DNA in pericentric heterochromatin for replication, which also makes this DNA accessible for transcription. Of no less interest is the next wave of pericentric transcription, which is confined to mitosis and characterized with smaller transcripts (~200 bp). Features of pericentric satellite transcription, specific for ontogenetic stages, cell ageing, cellular stress, cancers and other diseases have recently been reviewed [65,66,67]. These reviews consider features of histone modifications, DNA-protein interactions, and DNA methylation in pericentric heterochromatin.

More is known about the matter in fission yeast. Transcription in fission yeast proceeds during S phase of a cell cycle [71,72,73,74,75,76,77,78,79,80]. However, mechanisms of repeated DNA transcription and its regulation in mammals including repeats of Bs remain poorly studied. At least in some cases gene transcription in Bs might be a part of total heterochromatin transcription. It is also possible that by leading to a considerably increased share of heterochromatin in the genome, Bs can play a role in the regulation of both transcription of repeated sequences in A and B chromosomes and structural organization of chromatin in the nucleus. Unfortunately, our knowledge on transcription in Bs is limited. Data on Bs mostly derived from transcription analysis of a few isolated B chromosome genes and from gene composition of the Bs that were studied recently [31,32].

## 5. Where Do Mammalian B Chromosomes Reside in the Interphase Nucleus?

In interphase, mammalian chromosomes are not fixed at certain positions; however, in most cell types, chromosomal regions or even entire chromosomes show preference either at the internal or external compartment of the nucleus as well as to certain positions relative to the nuclear lamina and the nucleolus—depending on the number and transcriptional activity of the genes in these chromosomal regions or chromosomes [81,82,83,84]. C-positive regions are located in the nucleus peripherally and near nucleoli, usually in contact with the nuclear lamina. G-band material is also found closer to the nuclear periphery and the nuclear lamina, while R-band DNA most commonly is located in the internal compartment and makes no close contact with the nuclear lamina. The number of studies seeking to locate Bs in an interphase nucleus is not high. One worked with the raccoon dog and fox Bs [85], and another with Bs in fibroblasts and spermatocytes of the Korean field mouse [18].

The fox and raccoon dog Вs contained the *С-KIT* gene but had different sizes and preferences for locations in the interphase nuclei of the fibroblasts. Small-sized Bs of the fox were mostly found in the internal compartment of the nucleus, while their larger counterparts in the raccoon dog preferred peripheral locations. According to the authors [85], their data is consistent with the hypothesis that a chromosome is located in a nucleus depending on its size which is a statement from the chromosome size-dependent theory. However, a more detailed consideration indicates that this data is also consistent with evidence that transcriptionally active chromosomal regions show preference for the internal compartment of the nucleus, while transcriptionally inactive chromosomal regions for its periphery [83,86,87]. If a chromosome is small enough, this rule applies to the entire chromosome. A classic example is the contrasting manner in which human chromosomes 18 and 19, similar in size, are localized in the nucleus [88]. These chromosomes are contrasting in the number of transcriptionally active genes they contain. Chromosome 18 consisting mostly of G-bands is most commonly localized peripherally; while chromosome 19, mostly composed of R-bands, normally resides in the internal compartment. Localization data on canid Bs are difficult to interpret due to a lack of detailed information about DNA content in particular Bs. It is possible that small-sized Bs in the fox and medium-sized Bs in the raccoon dog have small transcriptionally active regions similar in size, and the difference in size that they still have could be due to a much larger heterochromatic block in the Bs of the raccoon dog. In this case, differences in the location of Bs can be accounted for by different ratios of transcriptionally active to transcriptionally inactive chromatin. It leaves no doubt that a detailed description of Bs appearing in the studies of interphase nuclear organization will be valuable for interpreting any relevant data obtained.

In a work analyzing the localization of Bs in ten the Korean field mouse specimens, all 103 Bs being studied were characterized by FISH with microdissected DNA probes, allowing the authors to assess the size of the regions composed of repeated sequences and the diversity of their repeats [18]. The number of Bs in a single individual’s cells varied from 3 to 19. The size, too, varied from a dot-like B to a large autosome. Regions homologous to particular chromosomal regions in the house mouse have been identified in two of these Bs [13], but no data on their transcriptional activity is available. In the fibroblasts, Bs were localized on the periphery of the interphase nuclei in associations with C-positive regions of A chromosomes. However, the distribution of the Bs in these associations was non-random and varied across individuals [18]. It is possible that the observed differences were due to different content of repeated sequences in the Bs that were present in the mice studied.

It is worth noting that the presence of supernumerary chromosomes leads to DNA increase in the genome. The amount of B material has been estimated to vary from 4% to 32% of the haploid genome of the Korean field mouse [18]. Furthermore, the additional material appeared as C-positive heterochromatin. In animals with a large number of macro-Bs, the amount of additional heterochromatin was substantially higher than the total amount of heterochromatin in A chromosomes [18]. Nevertheless, this variation of heterochromatin volume in the genome did not affect the normal function of the genetic machinery of the Korean field mouse cells in any way. The association of Bs with the C-positive regions of A chromosomes was likely to have helped preserve the architecture of the internal compartment of the nucleus, where most transcriptionally active genes reside. Apparently, the nucleus volume grew with the genome increasing [87,89,90]. However, because of denser heterochromatin folding, the former should have been increased to a lesser degree than the latter. Finding Bs on the nuclear periphery in associations with C-positive regions of A chromosomes is indicative of the need for an additional volume of the external compartment of the nucleus [18]. However, when it comes to change, the volume of the external compartment will be growing at a faster pace than that of the nucleus in its entirety. The ratio of the volumes of the external to the internal compartment can also be corrected for by a minor change of nuclear morphology. This correction is likely to take place naturally, because the formation of the nuclear envelope starts from the formation of the nuclear membrane on the heterochromatic regions of chromosomes [87,91]. 

In our opinion, the role of Bs in building the architecture of the interphase nucleus mostly depends on the composition of these chromosomes. Bs containing a small heterochromatic region and actively transcribed regions tend to have preference to the internal compartment of the nucleus, while the presence of large heterochromatic regions in Bs appears to promote their peripheral localization, in associations with heterochromatin of A chromosomes. This pattern of B chromosome localization will help maintain the optimal infrastructure of the internal compartment of the nucleus even if the number of Bs will be high.

## 6. Mammalian B Chromosomes: Origin and Evolution

**Figure 2:**
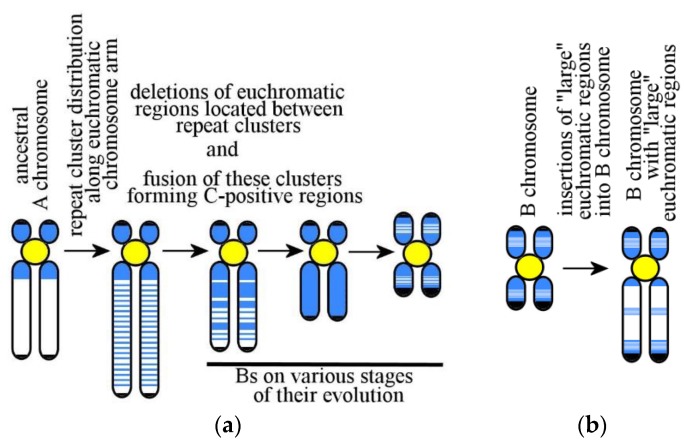
B chromosome arising through cluster repeats distributed along the euchromatic part of the chromosome arm of ancestral chromosome followed by the loss of the euchromatic region located between them (**a**) and Bs development through insertions of large euchromatic regions of A chromosomes or foreign DNA (**b**). Euchromatic regions and insertions of euchromatic material in Bs are white; heterochromatic regions and insertions of heterochromatic material in Bs are blue; active centromeres are yellow; inactive centromeres are brown; and telomeres on chromosome termini are black.

Addressing the question as to the emergence and evolution of Bs can be staged as follows: (*i*) Emergence of a B chromosome or its ancestor (proto-B chromosome); (*ii*) changes in its DNA content; (*iii*) its behavior in mitosis and meiosis; and (*iv*) action of natural selection. In theory, several scenarios can be proposed to explain the emergence of the B chromosome or its ancestor. Due to the structural similarities between mammalian Bs and human small SMCs, it is possible to suggest that the mechanism of their formation is also similar [43] (Figure 1). Additionally, it cannot be excluded either that Bs arose in result of a gradual degradation of the ancestral A chromosome [10] (Figure 2a) or developed through insertions of foreign DNA (Figure 2b).

If most proto-Bs really arose similarly to human small SMCs, it should be expected that Bs arose more frequently in the species with acrocentrics in their karyotypes. This tendency was earlier noticed [92,93]. The probability of a break in the long arm of an acrocentric chromosome is higher than that of two breaks at once in the proximal regions of two arms of a bi-armed chromosome. Moreover, the formation of a small SMC as an inverted duplication [43,94,95] (Figure 1) removes the problem of its protection from digestion with exonuclease. All these propositions make the suggestion of B chromosome formation mostly from acrocentric chromosomes very attractive. However, Bs were also found in species with karyotype containing mostly bi-armed chromosomes [7]. 

It is also possible that Bs may have arisen during interspecific hybridization, of which the role in speciation appears to be underestimated [96]. No proved case of mammalian Bs originated from interspecies hybrids has been published yet. However, we should keep in mind that traces of ancient interspecific hybridization could be erased during long time evolution. 

The possibility of the emergence of Bs by a gradual degradation of the Х-chromosome [10] and a gradual degradation of neo-Y chromosome [97] in grasshoppers make us consider one additional mechanism of B chromosome formation. The mechanism of gradual A chromosome degradation may, at least in part, be similar to that leading to small heterochromatic neo-Y-chromosome in some grasshoppers [97]. A hypothesis was put forward, which proposed the following sequence of events (Figure 2a): (*i*) Formation of the intercalary heterochromatic blocks in the euchromatic part of the original chromosome’s arm, (*ii*) reduction in the transcriptional activity of the euchromatic regions between heterochromatic blocks, (*iii*) increase of meiotic aberrations because of the presence of additional C-positive chromosomal regions, (*iv*) trisomy of this chromosome (one of the chromosomes with additional intercalary heterochromatic blocks) and (*v*) loss of euchromatic regions between heterochromatic blocks [97]. There is no described example of B chromosome formed by this mechanism, but it cannot be excluded that at least some mammalian Bs could have arisen in this way.

To add to this, there are mammalian Bs that consist mostly of C-negative regions [64]. Five Bs of yellow-necked mouse were studied by generating micro-dissected DNA libraries followed by their sequencing. Those Bs were large and composed predominantly of C-negative material. They contained C-negative regions showing G-banding [33] and DNA homologous to chromosomal regions in the house mouse, totaling a few millions of base pairs [13,31]. The organization of these chromosomes remains a mystery. They were devoid of regions homologous to extended regions of the reference genome but showed larger С-negative regions. In total, DNA in these Bs is homologous to about 3.5 Mbp of the euchromatic portion of mouse genome. It may be that they contain a large cluster of duplicons multiplied many times over. These chromosome regions might probably remain C-negative, despite the homology of their DNA to a relatively small portion of euchromatin in the reference genome. However, if this assumption is true, then they have not resulted from a gradual degradation of A chromosomes. Today, the suggestion on insertions into Bs rather large euchromatic regions from A chromosomes (Figure 2b) should remain among the hypothesis set about B chromosome origin and their further evolution. 

Data on B chromosome sequencing provided additional information on particularities of the B chromosome organization that requested for their explanation the development of the general theory B chromosome evolution. The sequencing of micro-dissected DNA libraries obtained from Bs demonstrated that Bs usually contain fragments of DNA from different A chromosomes [31], suggesting that most of them could be inserted into Bs at advanced stages of their evolution or could be included in the proto-Bs as duplicon cluster of an ancestral chromosome. To distinguish these two ways of B chromosome formation and further development, it is necessary to analyze DNA in regions between the centromere and the chromosomal hotspot breakpoint involved in the rearrangement provided proto-Bs. 

Analysis of homology between the pericentric regions of Bs and A chromosomes turned out to be intricate as well. In many species, pericentric heterochromatic regions contain—in addition to their specific sequences—a considerable number of sequences with homology to DNA in the pericentric regions of other chromosomes. The evolutionary rates of DNA in pericentric regions raise doubts as to the reliability of using these DNAs for identification of the origin of Bs [98,99,100]. These DNA markers might be reliable in some cases, but those cases are rare. For example, in the Korean field mouse, DNA sequences in the pericentric regions of autosomes and sex chromosomes are quite different [9]. FISH with microdissected DNA probes prepared from the pericentric region of an autosome painted the pericentric regions of all autosomes but gave no signal in the pericentric regions of the sex chromosomes. Most Bs of individuals from Siberian populations have in their pericentric regions DNA homologous to DNA in the pericentric regions of autosomes, while the Bs of specimens from the Russian Far East are devoid of this DNA in their pericentric regions [8]. It is possible that chromosomal rearrangement hotspots occur in different chromosomes in mice from Siberia and in mice from the Russian Far East. In the Siberian mice, they could occur in one or more autosomes; while in the Far East mice in a sex chromosome. The sequencing and a comparative analysis of Bs from inhabitants of these geographical regions could confirm their different origin and probably different ways of gaining even homologous sequences. Independent insertion of identical DNA fragments in Bs of different origin may be indicative of a high frequency of their transposition across the genome or positive selection in favor of the Bs that contain, such as DNA fragments. For example, detection of different margins of *Vrk1* region in Bs of two *Apodemus* species was considered as an indication to independent recruitment of the DNA fragments from the same genomic region [13].

Considering possible mechanisms by which Bs will be evolving, three points should be addressed: (*i*) changing of original DNA of proto-Bs; (*ii*) DNA amplification in Bs; and (*iii*) rearrangements involving large regions of Bs. Unfortunately, to date the sequencing of B chromosomal DNA has been performed for a small number of species and a small number of Bs [31]. Nevertheless, the differences between the homologous DNA insertions discovered in Bs of *Apodemus* mice captured from different populations suggested that the insertion of new DNA fragments from the main genome could take place also at advance stage of the B chromosome evolution [13]. 

**Figure 3:**
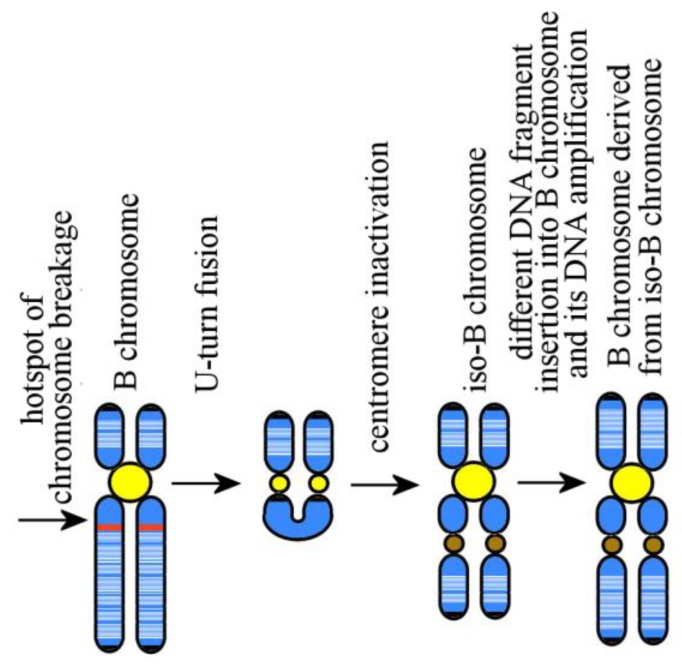
Iso-B chromosome formation through chromosome breakage at a hotspot of chromosomal rearrangements in a proximal region of a B chromosome followed by U-turn fusion, one centromere inactivation, and further B chromosome development. Euchromatic regions and insertions of euchromatic material in Bs are white; heterochromatic regions and insertions of heterochromatic material in Bs are blue; clusters of duplicons are red; active centromeres are yellow; inactive centromeres are brown; and telomeres on chromosome termini are black.

In most cases, Bs grow in size due to amplification of DNA fragments. The formation of amplicons and their amplification lead to the emergence of B chromosomal regions composed of tandem repeats [51]. This amplification may probably involve both repeated and unique DNA fragments of the main genome [31,51]. In the course of evolution, Bs may undergo rearrangements affecting their large regions. In a study on Bs in the Korean field mouse using FISH of microdissected DNA probes derived from individual Bs and their regions, regions along the chromosome arms were differentiated, and thus iso-Bs were revealed, and thus were Bs that have evolved from them [8,9]. The presence of iso-Bs in the karyotypes studied is consistent with the hypothesis stating that the proximal region of the ancestral A chromosome contained a chromosomal rearrangement hotspot and that more rearrangements involving the same hotspot occurred at more advanced stages of B chromosome evolution. Those later rearrangements led to an iso-B chromosome appearing in the form of an inverted duplication of one of its arms. The last rearrangement resembles the human small SMCs derived from the chromosome 15. Later on, differences in DNA amplification in the arms of this iso-B would lead to differences between these arms in size and DNA composition (Figure 3).

Analysis of a large number of the Korean field mouse specimens living in geographically well-spaced populations and displaying clear-cut differences in the sets of their Bs allowed the assessment of impact of known molecular genetic processes on evolution of the B sets in those populations [14]. We assumed that the observed differences in the morphology and DNA content of Bs is caused by different frequencies of chromosomal rearrangements in hotspots located in various chromosomes as well as variable intensities of some molecular genetic processes responsible for insertion of DNA fragments into proto-B chromosomes and amplification of their DNA like in formation of homogeneously staining regions (HSRs) [50,101]. Leaving open the question of whether natural selection has played a role in the formation of various sets of Bs in the Korean field mouse, we propose that different sets of Bs in isolated populations may have evolved without such selection. 

Analysis of mammalian B chromosome evolution must include an analysis the meiotic behavior of Bs. Analysis of the 3D organization of the nucleus in the Korean field mouse spermatocytes showed that Bs tend to be localized in the immediate vicinity of the sex bivalent [18]. These data agree well with the results of an analysis of chromosome spreads prepared from the cells at the pachytene stage [17,18]. The occurrence of Bs with both similar and contrasting DNA content could account for both paired and unpaired Bs in pachytene spreads. Some of the unpaired Bs, especially those without synaptonemal complex protein 1 (SCP1), were associated with asynapsed chromatin and the XY bivalent. The other unpaired Bs could form a separate association with asynapsed chromatin without the XY bivalent [17,18]. А and В chromosomes were distinguished in meiosis based on the absence or presence of phosphorylated histone H2A.X at different pachytene substages [18]. Some Bs still have H2A.X on them, while double-strand break repair in A chromosomes has been completed and phosphorylated histone H2A.X was not detected on them [18]. As is known, histone H2A.X phosphorylation is involved in meiotic sex chromosome inactivation (MSCI) and transcriptional silencing of unpaired chromatin (MSUC) in autosomes, which may lead to pachytene checkpoint activation and apoptosis [102,103]. However, it should be noted that a low degree of asynapsis can be ignored, as has been demonstrated for mice and humans [104,105,106,107,108]. It is possible that the pairing of Bs with each other or the pairing of different regions within a B chromosome and their association with the transcriptionally inactive XY bivalent allows the cell to avoid apoptosis and to accomplish meiosis. How Bs behave during female meiosis and whether their behavioral features may have impact on the probability of these chromosomes being transferred to an oocyte has yet to be elucidated.

## 7. Conclusions

A comparative analysis of B chromosomes in different mammalian species suggests that the emergence and evolution of these chromosomes must be due to a relatively small number of molecular and cellular mechanisms as well as some features of the main genome. They include different localization of chromosomal rearrangement hotspots in the proximal regions of chromosomes, proto-B chromosomes resulting from double-strand breaks at hotspots followed by errors in DNA repair, insertion of DNA fragments of the main genome into B chromosomes, and DNA amplification in B chromosomes. Differences in the frequency of these events and in the intensity of repair, transposition and amplification of DNA fragments result in different variants of B chromosomes, and their specific sets for particular species and even populations. When isolated from the main genome, DNA in B chromosomes may be evolving at increased rates. We do not exclude that natural selection favors В chromosomes with valuable genes, and although no evidence that genetic material moves from B chromosomes back to A chromosomes is available, it cannot be excluded.
